# Beyond the Operating Room: A Narrative Review of Enhanced Recovery Strategies in Colorectal Surgery

**DOI:** 10.7759/cureus.76123

**Published:** 2024-12-21

**Authors:** Hamed Ibrahim Hamed Albalawi, Rawshan Khalid A Alyoubi, Nawaf Mohsen Mubarak Alsuhaymi, Farha Abdullah K Aldossary, Alkathiry Abdulrahman Mohammed G, Fayez Mubarak Albishi, Jumana Aljeddawi, Fedaa Ahmed Omar Najm, Neda Ahmed Najem, Mohamed Mirza Ali Almarhoon

**Affiliations:** 1 General Surgery, King Fahad Specialist Hospital, Tabuk, SAU; 2 General Practice, Al-Rayan Colleges, Al-Madinah Al-Munawwara, SAU; 3 General Practice, Umm Al-Qura University, Makkah, SAU; 4 General Practice, King Faisal University, Al-Ahsa, SAU; 5 General Practice, Dammam Medical Complex, Dammam, SAU; 6 General Practice, Aseer Health Cluster, Abha, SAU; 7 General Practice, Ibn Sina Hospital, Makkah, SAU; 8 General Practice, Fakeeh College of Medical Sciences, Jeddah, SAU; 9 Surgery, Imam Abdulrahman Bin Faisal University, Dammam, SAU

**Keywords:** accelerated recovery, colorectal surgery, enhanced recovery after surgery (eras), pain management, postoperative care, postoperative complications

## Abstract

Enhanced Recovery After Surgery (ERAS) protocols have significantly transformed the management of patients undergoing colorectal surgery. This comprehensive review explores the key components and benefits of ERAS in colorectal procedures, focusing on preoperative, perioperative, and postoperative strategies aimed at improving patient outcomes. These strategies include preoperative patient education, multimodal analgesia, minimally invasive surgical techniques, and early mobilization. ERAS protocols reduce postoperative complications, shorten hospital stays, and enhance overall recovery, leading to better patient satisfaction and decreased healthcare costs. However, challenges such as patient adherence and managing high-risk patients remain critical areas for further research. Additionally, future research should focus on refining ERAS protocols, integrating novel technologies such as minimally invasive techniques, and evaluating long-term outcomes to further enhance the recovery process.

## Introduction and background

Colorectal cancer (CRC) is a type of cancer that affects the colon (large intestine) or rectum. It is one of the most common types of cancer worldwide. The risk of CRC increases with age. Most cases affect people over 50 years old [1]. We perform colorectal surgeries to repair a damaged or diseased section of the large intestinal tract. This may be a result of cancer, a malformation, another disease process, or some sort of injury or infection. Planning colon resection needs to take into account the nature of the lesion and its location within the colon [2]. Colorectal surgery has been linked to an increase in hospital length of stay (LOS) and a high incidence of surgical site infections (SSIs) [3,4]. In addition, the incidence of perioperative nausea and vomiting (PONV) within hospitals may approach 80%, and readmission rates may approach 35% [5].

Enhanced recovery protocols (ERPs) are a collection of standardized perioperative procedures that are administered to patients undergoing elective surgery. They may change greatly depending on the condition of the cases. These programs integrate patient education, perioperative management protocolization, and the employment of a multidisciplinary team [6]. Although these protocols are generally not meant for non-elective cases, urgent and emergent patients could benefit from some of the ERPs' components [6,7]. ERPs, sometimes called fast-track procedures or Enhanced Recovery After Surgery (ERAS), are intended to enhance patient outcomes. Early bowel function recovery, reduced wound infection rates, and a shorter LOS are among the intended outcomes [8].

The ERAS protocols are used to overcome the limitations of the traditional recovery methods. These traditional methods include routine fasting until bowel function returns, limited ambulation during the early postoperative period, and the use of nasogastric tubes and drains. Patients were traditionally kept nil per os (NPO) until bowel sounds returned, delaying oral intake. This practice has been shown to prolong recovery and delay nutritional rehabilitation [9,10]. Early ambulation was not routinely emphasized, leading to prolonged immobility, which increased the risk of venous thromboembolism (VTE), deconditioning, and slower recovery of normal gastrointestinal (GI) function [9]. Routine placement of nasogastric tubes and surgical drains aimed to reduce complications like anastomotic leaks and bowel obstruction. However, studies have demonstrated that these interventions may not improve outcomes and can lead to patient discomfort and increase LOS [11]. Opioids were the cornerstone of pain management but contributed to delayed bowel function, respiratory complications, and opioid-related side effects, including dependence [9]. Traditional methods lacked a structured, multidisciplinary approach to perioperative care, often resulting in fragmented care and inconsistent application of best practices [11]. 

These limitations have driven the development of ERAS protocols, which emphasize early feeding, multimodal analgesia, and early ambulation, significantly improving postoperative outcomes. Studies have shown that ERAS protocols reduce LOS in comparison to traditional recovery methods, decrease postoperative complications, and improve overall recovery without compromising safety [11,12]. Additionally, ERAS protocols enhance patient satisfaction by fostering a faster return to normal activities and minimizing discomfort. Although many of the ERPs' components might be regarded as standard care [13], there are still obstacles standing in the way of the program's complete implementation [14], despite recommendations from health systems [15]. In addition, the best ways to speed up recovery and improve patient outcomes are still unclear. Furthermore, less is known about the contribution of more recent modalities to the optimization of recovery, such as prehabilitation, multimodal analgesia, and minimally invasive surgical (MIS) methods. This gap includes the requirement for more comprehensive data on how these treatments affect patient-centered outcomes like long-term complication rates, functional rehabilitation, and quality of life (QoL).

Therefore, we perform this review to discuss the current evidence and best practices related to optimizing recovery following colorectal surgery outcomes to provide a comprehensive understanding of current preoperative, perioperative, and postoperative strategies and outcomes related to colorectal surgery. 

## Review

Methods 

Between 2014 and 2024, searches were conducted using the following terms in the National Institute of Health's PubMed/MEDLINE, Scopus, and Web of Science databases: "Colon and Rectal Surgery" OR "Colon Surgery" OR "Rectal Surgery" OR "Colorectal Surgery") AND ("enhanced recovery" OR "postoperative care" OR "recovery optimization" OR "accelerated recovery") AND ("length of stay" OR "postoperative complications" OR "pain management" OR "postoperative ileus" OR "patient satisfaction"). We focused on studies discussing the current evidence and best practices related to optimizing recovery following colorectal surgery outcomes to provide a comprehensive understanding of current practices and outcomes related to colorectal surgery. This could involve evaluating the effectiveness of ERAS protocols, the role of preoperative interventions such as prehabilitation and MIS, and approaches to pain management and nutrition.

ERAS protocols

This clinical practice guideline was produced in collaboration between the Society of American Gastrointestinal and Endoscopic Surgeons (SAGES) and the American Society of Colon and Rectal Surgeons (ASCRS). The goal of the combined ASCRS/SAGES committee is to provide the highest standard of care available today for improved recovery following colon and rectal surgery. This guideline is not restrictive; rather, they are all-inclusive [16]. The purpose of these guidelines is to provide the best available options regarding the management of cases undergoing colorectal surgery. It provides this information to patients, healthcare professionals, and practitioners. The outcomes of interest are improved wound healing, early hospital discharge, early restoration of bowel function, and freedom from nausea and pain during rest [17]. 

A typical ERP consists of numerous preoperative, perioperative, and postoperative components. Because these components are typically applied simultaneously, it can be challenging to determine which of the bundles of measurements is most useful. The greatest indicators of a shorter LOS, based on a retrospective analysis of eight years of ERP compliance, were early mobilization, non-opioid analgesia, early oral nutrition (early stopping intravenous fluids), the removal of a nasogastric tube, and early epidural and urine catheter removal [18].

Preoperative Interventions 

A patient's suitability for surgery, the type of treatment to be administered, the anticipated postoperative site, and the type of anesthesia to be used can all be assessed by medical professionals during the preoperative phase [19]. Among the interventions that occur during this period are preoperative education, adherence to preoperative fasting recommendations, bowel preparation, and routine VTE, antiemetic, and antimicrobial prophylaxis.

Preadmission counseling: Preoperative education is necessary to assist patients and their families in setting reasonable expectations for their experiences with surgery, anesthesia, and postoperative pain management [20]. Patient education should also include how to deal with stoma, which is known as an important cause of prolonged LOS. A single-center prospective study was conducted to ascertain whether an ERAS program incorporating counselling and stoma education can decrease hospital stays, decrease readmissions, and enhance quality of life. In comparison to the standard treatment group, the ERAS group with education had a significantly shorter total hospital stay (p<0.001). The two therapy groups showed comparable results in terms of overall major and minor morbidity, QoL, readmission rate, problems linked to the stoma, and 30-day mortality [21]. 

In order to prevent pneumonia and promote early oral intake, the preoperative procedure uses incentive spirometry to improve postoperative atelectasis. It also includes counseling about early mobilizing to accelerate their postoperative progress and prevent deep venous thrombosis [22]. Long-term immobility can cause several problems, including insulin resistance, thromboembolic disease, skeletal muscle loss and weakness, and reduced ability to exercise. For each day of bed rest, muscle mass is thought to decline by 1.5-2% [23].

Optimizing the patient before surgery, including the management of comorbid conditions such as hypertension, diabetes mellitus, coronary heart disease, anemia, and chronic obstructive pulmonary disease (COPD), is a significant component of preoperative management. Additionally, patients receive education on how much alcohol and smoke they consume. ERAS procedures also emphasize several patient-optimization techniques, such as educating patients about proper sleep hygiene, relaxing techniques to lessen anxiety, and making nutritional adjustments [24]. 

Preadmission nutrition: Although patients must fast for eight hours before surgery, it has been recommended that they maintain hydration and shorten the fasting duration by consuming clear fluids up to two hours before the surgery. Two hours before surgery, allowing patients to consume a clear carbohydrate drink helps to keep their physiological state as close to normal as possible. It has been demonstrated that consuming a high-carb diet helps lower insulin resistance, maintain muscle mass and lean body mass, speed up the restoration of intestinal function, and lessen discomfort and anxiety in patients [25]. 

Around 1.2-1.5 g/kg/d of protein with oral dietary supplementation throughout one to two weeks is supported by numerous national and international standards. Malnourished individuals undergoing elective GI surgery have been associated with reduced complications that may happen after surgery when using this technique [26,27]. Supplements that contain immune-modulating nutrients such as nucleotides, glutamine, arginine, and fish oil (ω-3 fatty acids) are known as immunonutrition supplements. The superiority of immunonutrition over traditional high-protein oral dietary supplements is still up in controversy. Preoperative immunonutrition, on the other hand, has been associated with a shorter LOS and fewer complications, including infectious issues [28,29].

Mechanical preparation and preoperative oral antibiotics: Another approach to preoperative protocols is mechanical bowel preparation (MBP). In 2011, a Cochrane review of randomized controlled trials (RCTs) reported that anastomotic leaks or problems following colorectal surgery were not mitigated by MBP alone [30]. Based on a meta-analysis of seven RCTs with 1769 patients that compared MBP+oral antibiotics with MBP alone, there was a decrease in overall SSIs (p<0.001) and incisional site infections (p<0.001), but the rate of organ/space infection following elective colorectal surgery did not differ significantly [31]. Additionally, MBP combined with oral antibiotic preparation after left colon resection was linked to lower rates of superficial SSIs, intra-abdominal infections, overall morbidity, and anastomotic leaks, according to a retrospective analysis of a national database from the United States [32]. Preoperative *Clostridium difficile* colitis and SSIs were less common in patients who received MBP and oral bowel preparation than in those who did not get bowel preparation, according to data from the Michigan Surgical Quality Collaborative database [33]. Therefore, for elective colorectal surgery, the ASCRS 2019 Clinical Practice Guideline on Bowel Preparation recommends utilizing an MBP in addition to oral antibiotics before surgery [34].

Preadmission rehabilitation: Improving the patient's condition before surgery, also known as prehabilitation, has been proposed as a possible strategy to enhance outcomes following surgery [35,36]. Prehabilitation enhances physical function before major abdominal or colorectal surgery, according to the most recent clinical studies [37-42]. The outcomes of the most recent systematic reviews are in line with these findings [43-46]. Following major abdominal surgery, patients who had prehabilitation had considerably decreased rates of pulmonary, cardiac, and overall problems, according to a meta-analysis of 35 trials [47]. Eight trials with 442 patients undergoing significant liver, GI, colorectal, and abdominal procedures were included in the meta-analysis. Comparing the prehabilitation group to the control group, it was found that there was no significant difference in LOS and a substantial decrease in postoperative pulmonary complications (PPCs) and total postoperative morbidity [48]. Prehabilitation may be especially beneficial for patients undergoing open surgery who have lower baseline functional ability, even though the current data are still restricted due to numerous underpowered trials [38-41].

Perioperative Interventions 

SSI control: The perioperative protocols for colorectal surgery involve the administration of multimodal non-opioid analgesics and antiemetics, along with a combination of lung-protective mechanical ventilation, euvolemia, regional anesthetics, antibacterial prophylaxis, and normothermia. It also entails reducing the use of tubes and drains, such as Foley catheters, nasogastric tubes, and surgical drains that are removed early. Numerous hospitals nationwide have already implemented thromboembolic prophylaxis and SSI prevention strategies, which are now part of ERAS procedures [49]. Using chlorhexidine alcohol skin wipes, changing gloves and instruments, irrigating them with antibiotics before closure, administering intravenous antibiotics before surgery, and taking antibiotics along with bowel preparation are some of the preventive measures for SSIs [50-54]. A meta-analysis included 17,557 patients to detect the SSI preventive packages. Risk reductions of 40% were noted in the incidence of SSIs overall, 44% in the rate of superficial infections, and 34% in the rate of deep and organ space infections. Additionally, it stated that the most important procedures to follow to lower the incidence of SSIs were the use of sterile wound closure trays, MBP with oral antibiotics, and glove changes before fascial closure [55]. Another meta-analysis involving 20,701 patients found that while compliance rates and component aspects of the SSI reduction bundle varied significantly (from 19% to 90% in the included studies), the odds ratio of SSIs was 0.56 when a bundle was used in comparison to the control group [56]. There is consistent evidence linking SSI preventive bundles with higher rates of compliance with particular bundles to significantly lower the incidence of SSI.

Pain management: Other strategies include providing appropriate pain management [49]. It is also recommended to minimize the use and the dose of opioids to avoid ileus after surgery. Reduced opioid use following colorectal surgery has been linked to a quicker recovery of bowel function and a shorter LOS [4,57]. To reduce the use of opioids and provide appropriate pain management, the ERAS protocols recommend the use of multimodal analgesia such as nonsteroidal anti-inflammatory drugs (NSAIDs), acetaminophen, gabapentinoids, α2-agonists, magnesium, lidocaine, and ketamine when necessary. Recent clinical studies and meta-analyses have demonstrated that NSAIDs may increase the risk of anastomotic leak [58-60]. This possible impact on anastomotic leak appears to be molecule- and class-specific, as further studies have shown [61]. In a retrospective cohort performed on patients undergoing elective colorectal surgery, the risk of anastomotic leak rate was 11.8% versus 6% (p=0.01) in patients using diclofenac; leak rate differences with other NSAIDs were not seen [62]. Moreover, two meta-analyses have demonstrated that while taking some NSAIDs (such as cyclooxygenase 2 inhibitors) does not increase the risk of anastomotic leak, consuming NSAIDs in general does. Anastomotic leak was not linked to the use of ketorolac or selective NSAIDs in these trials, while nonselective NSAID diclofenac use was linked to a higher leak risk (p<0.001) [58,59]. 

The use of gabapentinoids is controversial because two large database studies found no decrease in postoperative opioid intake and a rise in pulmonary problems following colorectal or orthopedic surgery [63,64]. In addition, a meta-analysis assessing the perioperative use of gabapentinoids found little evidence of a clinically meaningful analgesic effect and concluded that routine use of these drugs was not advised [65]. For individuals who have chronic pain, a low-dose ketamine infusion administered after surgery can be particularly helpful [66,67]. Nevertheless, its associated sedation, vertigo, and psychotropic side effects could hinder a quick recovery, especially in older individuals [67]. In addition, magnesium can be a helpful adjuvant and is also linked to a reduction in postoperative opioid intake when given as a bolus or infusion [68].

Furthermore, regional anesthetics and surgical field blocks are adjuncts that can be utilized to relieve pain during and after surgery. Regional anesthesia, such as continuous or single-shot peripheral nerve blocks, and neuraxial anesthetic techniques can reduce opioid use during open and laparoscopic colorectal surgery [68,69]. A growing number of block options are available, including rectus sheath, erector spinae, quadratus lumborum, and transversus abdominis plane (TAP) blocks. Two meta-analyses using TAP blocks demonstrated decreased LOS following laparoscopic colorectal surgery as compared to systemic opioid administration [69,70]. To manage pain during MIS, laparoscopic-guided TAP block is both safe and effective, according to a recent systematic review and meta-analysis. It seems to be equally effective as ultrasound-guided TAP blocks in reducing postoperative opioid use and controlling pain at the outset of recovery [71]. In the perioperative context, intrathecal morphine injection combined with spinal analgesia is an additional option. According to recent trials and meta-analyses, intravenous opioids are less effective than intrathecal morphine during laparoscopic surgery, and intrathecal morphine is related to lower pain levels [4,72,73]. 

A multimodal pain management approach considers the patient's medical history and coexisting conditions, the type of surgery and anticipated pain following it, the emotional and psychological status of the patient, their baseline pain threshold, and the advantages and disadvantages of the various medications [74,75].

Table 1 summarizes the efficacy and safety of the most important analgesic drugs.

**Table 1:** Summary of the most important analgesic drugs' efficacy and safety. GI: gastrointestinal; LOS: length of stay; NSAIDs: non-steroidal anti-inflammatory drugs

Analgesic drug	Efficacy	Adverse effects	References
NSAIDs (ketorolac/diclofenac)	Effective in reducing postoperative pain and opioid consumption.	Risk of GI bleeding, renal impairment, and potential for anastomotic leak in bowel surgery. Risk of GI bleeding, renal dysfunction, and delayed wound healing. Risk of cardiovascular events, GI bleeding, and renal impairment.	[58-60]
				Opioids	Effective for moderate to severe pain control; cornerstone in perioperative analgesia.	Risk of respiratory depression, constipation, ileus, and increased hospital LOS.	[4,57]
				Gabapentinoids (gabapentin/pregabalin)	Effective for neuropathic pain and can reduce opioid consumption when used preoperatively.	Drowsiness, dizziness, risk of sedation, and potential respiratory depression when combined with opioids.	[63-65]
Magnesium	Enhances opioid analgesia and may reduce postoperative pain and muscle spasms.	Risk of hypotension, bradycardia, and hypermagnesemia with high doses.	[68]
Ketamine	Effective for reducing postoperative pain, opioid-sparing effects, and chronic pain development.	Risk of hallucinations, delirium, and potential for increased heart rate and blood pressure.	[66,67,76]

PONV control: PONV frequently results in both a prolonged hospital stay and decreased patient satisfaction. Female, younger age, non-smoking status, history of motion sickness, history of PONV, laparoscopic surgery, an extended duration of surgery, volatile anesthetic, and opioid analgesia are considered risk factors [77]. There are multiple perioperative protocols to prevent PONV. Preoperative and intraoperative prophylactic antibiotic therapy has been proven to be highly effective in controlling PONV. The anesthesia team can determine what causes nausea and vomiting and take appropriate action. Using regional anesthesia or propofol-based complete intravenous anesthesia, avoiding volatile anesthetics, and utilizing multimodal analgesia to minimize perioperative opioids are some strategies to lower the incidence of PONV. Additionally, using combination antiemetic medication as a preventative measure, such as dimenhydrinate before surgery, dexamethasone, and ondansetron during it, can be highly successful in controlling PONV [78-80]. For individuals who are at high risk of PONV, a transdermal scopolamine patch is advised [49].

Multimodal PONV prevention is linked to lower risks of PONV and readmission following colorectal surgery [81-83]. Combination treatment of two or more antiemetics is superior to taking a single medication in preventing PONV [84-88]. When used in conjunction with other antiemetics, dexamethasone significantly improved PONV prophylaxis compared to antiemetics alone and decreased the requirement for rescue medication [89]. Furthermore, some meta-analyses revealed that dexamethasone had no appreciable effect on glycemic control or a rise in postoperative infections [89,90].

Postoperative Interventions 

Early mobilization: As we mentioned before, patients in the preoperative stage are counseled about the importance of early mobilization to prevent postoperative atelectasis. Physical activity can mitigate or prevent the deconditioning associated with bed rest [18,91]. Following colorectal surgery, early ambulation has resulted in quicker recovery and fewer problems [92-94]. A shorter LOS was associated with a higher step count on the first postoperative day (POD) after thoracic or major abdominal surgery in a prospective cohort study involving 100 participants [95].

A recent systematic analysis found that there aren't enough RCTs to support this suggestion [96]. A recent RCT reported that early mobilization didn't influence many outcome indicators, like the return of functional walking ability after four weeks, GI function recovery, duration needed to meet 30-day total complications, and discharge requirements, as well as the patient-reported outcome metrics. The only side effect linked to early mobilization is intolerance to orthostatic hypotension [97]. Another RCT of a priori secondary outcomes was done on patients undergoing colorectal surgery to calculate the degree to which staff-directed early mobilization facilitation affects 30-day PPCs and the recovery of pulmonary function after colorectal surgery. Staff-directed early mobilization facilitation did not reduce PPCs or improve postoperative pulmonary function in an optimal recovery pathway after colorectal surgery. Thus, to improve pulmonary outcomes, there is no need to devote more resources (staff time) to promoting early mobilization [98].

Ileus prevention: In a Cochrane systematic review of 17 RCTs comparing "late initiation" following lower GI surgery and early initiation of feeding (i.e., within 24 hours of surgery), it was reported that early feeding was linked to a two-day reduction in hospital stay duration [99]. Nonetheless, the care approaches of the perioperative clinical trials differed greatly. The reported mean LOS in the control group varied from six to 24 days. Moreover, early feeding did not affect the likelihood of complications, including anastomotic leak, wound infection, pneumonia, or mortality. In this review, there was no statistically significant increase in symptoms of nausea and vomiting among the early feeding group. Early enteral feeding is linked to the early recovery of GI function as well as a shorter time to flatulence and the first bowel movement [100]. The majority of the evidence points to the advantages of early feeding, despite trial heterogeneity.

It has been proposed that sham feeding, such as chewing gum, speeds up the recovery of GI function by stimulating the vagal cholinergic system, which in turn speeds up bowel movements [101]. Chewing gum following colorectal surgery has been examined in a meta-analysis of 18 RCTs [102]. In most of these trials, participants chewed sugar-free gum for three minutes, three times a day. Nonetheless, the vast majority of these RCTs were low-quality and highly susceptible to bias. Chewing gum was linked to a shorter time to first flatus, a shorter time to first bowel movement, and a decrease in LOS [102]. Additionally, the chewing gum arm had a decreased pooled result of postoperative ileus. There was no significant difference in other outcomes, such as complications, readmission, or reoperations, between the two groups. A subgroup study of open and laparoscopic procedures upheld these noteworthy correlations. Chewing gum was no longer linked to significantly shorter times to flatus and LOS, according to a subgroup analysis of trials conducted in the framework of an ERP [103].

An early first flatus and bowel movement, as well as a lower incidence rate of postoperative ileus, were associated with chewing gum without influencing LOS in another systematic review and meta-analysis that included just 10 high-quality RCTs [104]. The majority of research indicates that chewing gum is safe and inexpensive and it may only have a minor impact on GI recovery without a significant impact on LOS.

Coffee may even help the GI tract heal more quickly following colorectal surgery, according to certain evidence [105,106]. According to a meta-analysis of seven RCTs involving 606 patients, coffee and caffeine can stimulate the lower GI system and maybe lessen postoperative ileus. Coffee consumption shortens the time to first bowel movement and oral intake tolerance, but it does not affect the time to flatus, overall problems, or LOS [107].

Postoperative follow-up: ERP typically results in a shorter hospital stay and, within the context of post-surgical risk management, raises the issue of postoperative surveillance at home. Establishing a postoperative surveillance system is crucial when patients are contacted by a nurse the day following surgery, similar to an ambulatory surgery [103].

**Figure 1:**
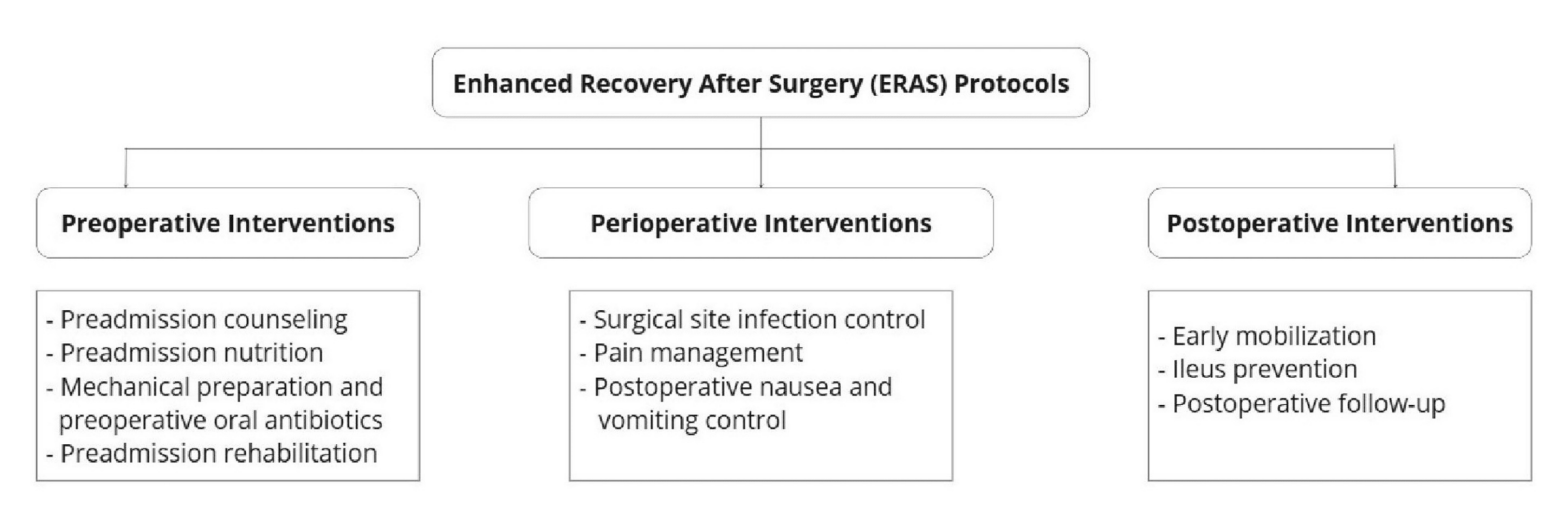
Summary of the steps of ERAS. Image Credit: Authors ERAS: Enhanced Recovery After Surgery

Texting is an effortless and quick way to stay in touch with someone after surgery. According to the French Regulatory Authority of Electronic and Postal Communications (ARCEP), 79.9 million subscriber identity module (SIM) cards were in use in France in late 2014 [108]. The usefulness of text messages (TM) has been demonstrated in several medical contexts. TM is used to effectively remind patients of upcoming appointments for medical care [109] and so promote adherence to the therapy [110]. In a prospective multicenter trial to assess, within an ERP, the viability of TM in conducting home surveillance following colorectal surgery, patients were asked, on the first, third, and fifth days following discharge, four brief questions about pain, bowel movements, fever, and phlebitis. An automated alarm was transmitted over the Internet to the attending physician in the event of an irregular or non-responsive response, and the patient was notified right away. This trial reported that 90% of patients replied to every TM. There were 48 alarms, with 56% being from discomfort and 40% from the TM not being responded to. Four percent of patients received in-hospital care as a result of alerts, including two unscheduled reoperations and three rehospitalizations. On a scale of 1-5, the median satisfaction score was 5, with 85% of patients responding. These results raise the idea of using text messaging for post-discharge home surveillance for patients receiving colorectal surgery inside an ERP, similar to ambulatory surgery [111]. All steps of ERAS are summarized in Figure 1.

New surgical techniques 

Laparoscopy used in colorectal surgery is supported by strong evidence. For the results of short-term results such as LOS, blood loss, and postoperative pain, the use of laparoscopy is better than open resection in two distinct multicenter RCTs involving patients with colon cancer. The COlorectal Cancer Laparoscopic or Open Resection (COLOR) study was conducted in the Netherlands, and A Randomized Controlled Trial Comparing Quality of Life Following Laparoscopic Versus Open Colectomy for Colon Cancer (ALCCaS) trial was conducted in Australia [112,113]. Comparing laparoscopic colonic resection to open resection, other RCTs have reported a decrease in perioperative morbidity, including nonsurgical, wound, and overall morbidity [114,115]. These outcomes are in line with extensive database studies that supported the use of laparoscopy and used information [116,117].

Over the past 10 years, robotics' use in colorectal surgery has grown dramatically [118], and numerous trials have shown that robotics for colorectal surgery is both feasible and safe [118-122]. However, the advantages of the robotic technique over traditional laparoscopy in terms of immediate- and long-term surgical results are still unclear. RCTs and meta-analyses indicate reduced conversion rates when using a robotic approach [118,120-122]. Until now, operative durations and expenses are typically greater when using robotic surgery, and the rates of complications are comparable with the results of laparoscopy [119,121]. 

The combination of MIS and an ERP yields the best results; this was shown in the four-arm perioperative strategy in colonic surgery: LAparoscopy and/or FAst track multimodal management versus standard care (LAFA) trial. In this experiment, the patients with the lowest LOS and morbidity were those who underwent laparoscopic surgery inside an ERP [123]. Therefore, an MIS is advised to maximize postoperative healing inside an ERP.

Discharge criteria 

After colon surgery, standard discharge criteria include bowel function returning, oral intake tolerance, appropriate pain management with oral analgesics, and the ability to move around without any difficulties [124]. By POD 1 or POD 2, many patients fulfill these requirements [125]. Nonetheless, there are more reports of same-day discharge; that depends on whether it is possible to release patients before their bowel function returns. 

More than 10 years ago, small case series were the first to report on the idea of the "ambulatory" or "outpatient" colectomy [126]. This early study describes the effective discharge of low-risk patients following colorectal resection at home following a 24-hour surveillance period without unwarranted problems [126]. In an RCT of patients receiving MIS of colon resection for cancer, 30 patients were randomly assigned to receive conventional postoperative treatment with discharge following the restoration of bowel function or to be discharged on POD 1, regardless of intestinal function, with telemedicine follow-up on POD 2 [127]. Adverse events and QoL did not differ between the two groups in this trial (p=0.041).

Some clinical studies reported lower readmission rates associated with same-day release following colorectal surgery [31,128,129]. One of these studies, which is a retrospective study, reported that 93% of patients may be discharged the same day, with a 6% readmission rate [129]. This experiment discovered that for some patients with acceptable complication rates, same-day discharge within an ERP is possible [129]. There is little, but growing, evidence in this field. Suggestions may vary when new data becomes available.

Barriers to optimizing recovery

Oral consumption before surgery results in canceling cases in some conditions. Some procedures may need to be canceled because some patients may not have completed the two hours of liquid fasting and the six hours of solid meal fasting that are required by recommendations for their surgery to proceed. As a result, several facilities have decided to increase the recommended solid fasting period to eight hours and advise patients to refrain from drinking anything three to four hours before the planned procedure. Nevertheless, if you keep fasting after this point, you increase the danger of developing hypoglycemia and elevated insulin levels [130]. 

The surgical team may encounter difficulties due to the scheduling of drugs. According to a trial on the prophylaxis of VTE in gynecologic oncology, starting heparin doses before surgery reduced the incidence of deep vein thrombosis following surgery as well as the number of deaths associated with the condition [131]. Nevertheless, American and European anesthesia guidelines recommend starting preventive subcutaneous heparin intraoperatively one to two hours after the epidural is placed. Less than 10 documented cases of bleeding for prophylactic dosages served as the basis for this suggestion. Additionally, it is recommended that epidural catheters be placed or taken out no earlier than 12 hours following the previous low-molecular-weight heparin dose or two hours following the last unfractionated heparin dose [132,133]. So each surgical case should be carefully evaluated to determine whether a spinal anesthetic or epidural is necessary.

Many ERAS guidelines recommend starting a regular diet as early as the evening after surgery. On the other hand, forcing people to eat will not help them to recover early [134]. Patients must understand that they can eat as long as the food is sufficiently tolerated. Patients who are ordered to exercise as tolerated are unlikely to result in an acceptable early mobilization unless there is a committed team to monitor and support the exercise and enough staffing to ensure that pain, nausea, and vomiting are under control. As many surgical units have found, increasing the number of assistants and nurses on staff is required to ensure early mobilization [96,97]. 

Cost-effectiveness of the implementation of ERAS

Reducing hospital stays, encouraging patients to regain mobility and function, and lowering the incidence of problems following surgery are all goals of better recovery, which may also result in lower medical expenses. An ERAS strategy may save US$2200-2500 for each patient treated, according to cost data gathered from trials performed on patients who had gynecological and colorectal surgeries to examine the enhanced recovery techniques [135,136]. According to the results of their economic analysis, the ERAS multi-site colorectal surgical program in Alberta saved $3.8 (a range of $2.4-5.1) for every $1 invested in the procedure [137]. To continue evaluating the true impact of cost and duration of stay, more ERAS protocol review and implementation are required [138].

Future directions and innovations

A multicenter prospective cohort study called the POWER study involved 2,084 patients who were scheduled for elective colorectal surgery. According to the findings, patients with higher adherence rates had lower rates of mortality, overall complications, and moderate-to-severe consequences than patients with lower adherence rates [138]. Thus, the question is how to create and manage an ERAS program that is broadly acceptable in clinical practice with just modest restrictions. Certain ERAS interventions have less than 50% compliance, as demonstrated by Toh et al. [139]. So, we must assess evidence of compliance for low-compliance elements, including diet progress and pain management, to create an ERAS program that is more useful for actual clinical practice. Furthermore, achieving the main goal of the ERAS program for colorectal surgery required an indicator of program application.

Koh et al.'s study examined the ERAS program's indications and assessed the viability and safety of using the ERAS protocol on elderly patients with CRC [139]. There are now more older people with CRC due to an aging population. Older patients require additional care and a multimodal strategy to decrease postoperative morbidity and enhance recovery since they are physiologically more fragile, have poorer performance status, and have more comorbidities. As a result, it is imperative to assess the ERAS program's effectiveness and safety for use with the elderly. The authors compared the perioperative outcome and LOS between the old group, which consisted of patients over 70, and the young group. Between the elderly and younger groups, there were comparable LOS and postoperative problems. There was no correlation between advanced age (>70 years) and a significant rate of surgical complications. Consequently, the authors came to the safe and workable conclusion that patients older than 70 years old can safely follow the ERAS regimen. The authors assessed that the ERAS technique may be used even for individuals with certain problems, such as the elderly. To enhance surgical results and compliance, the ERAS protocol should be developed taking cohort factors like age, comorbidity, or preoperative performance status into account [140].

A common application in colorectal surgery is the MIS. Because MIS and ERAS shared the benefit of reduced postoperative pain and recovery, the MIS would help the ERAS program be adopted in clinical practice. Given how widely MIS is used in colorectal surgery, the ERAS would spread quickly. Thus, this is the right moment to suggest the ERAS program's future course in colorectal surgery [141]. 

Limitations 

One of the most important limitations of our study is that it discusses the most recent published trials narratively and did not follow the strict protocols of systematic reviews, which can introduce selection bias. The choice of included studies and sources may not comprehensively represent all available evidence. In addition, the qualitative nature that was used to analyze the available data can lead to subjective interpretation and synthesis of the literature, potentially overlooking conflicting evidence. Insufficient published quantitative data limits statistical analysis, restricting the capacity to derive high-certainty-level findings regarding effect sizes or comparative efficacy. Moreover, without a structured approach to study selection and critical appraisal, the findings might not be as broadly generalizable compared to systematic reviews.

A narrative review was chosen to allow for a broader exploration and contextual discussion of enhanced recovery strategies, which may span diverse topics beyond those easily categorized or quantified. This approach provides flexibility to address emerging themes, expert opinions, and practical applications that might not fit within the constraints of a systematic review.

## Conclusions

The integration of multimodal analgesia and MIS techniques is particularly effective in improving pain control and functional recovery, reducing reliance on opioids, and enhancing patient satisfaction. Implementing evidence-based interventions has demonstrated clear benefits in reducing morbidity, accelerating recovery, and minimizing costs. While the positive impact of ERAS is well documented, there are ongoing challenges to optimizing the recovery. Future research should focus on refining ERAS protocols, integrating novel technologies such as minimally invasive techniques, and evaluating long-term outcomes to further enhance recovery. 
